# International Journal of Infectious Diseases: from the past quarter-century to the next

**DOI:** 10.1016/j.ijid.2021.06.064

**Published:** 2021-07-01

**Authors:** E. Petersen, S.S. Lee, L. Blumberg, L.D. Kramer, C. Obiero, S. Al-Abri, A. Abubakar, T.C.A. Pinto, B.R. Yapi, P.A. Tambyah, A.H. Holmes

**Affiliations:** International Society for Infectious Diseases, Boston, United States. Institute for Clinical Medicine, Aarhus University, Denmark; International Society for Infectious Diseases, Boston, United States. Stanley Ho Centre for Emerging Infectious Diseases, Faculty of Medicine, The Chinese University of Hong Kong, Shatin, Hong Kong, China; International Society for Infectious Diseases, Boston, United States. Centre for Emerging, Zoonotic and Parasitic Diseases, National Institute for Communicable Diseases, Johannesburg 2195, South Africa; International Society for Infectious Diseases, Boston, United States. School of Public Health, State University of New York at Albany, Albany, NY, USA; International Society for Infectious Diseases, Boston, United States. Clinical Research Department, KEMRI-Wellcome Trust Research Programme, Kilifi, Kenya; Department of Global Health, University of Amsterdam, Faculty of Medicine, Amsterdam, Noord-Holland, The Netherlands; International Society for Infectious Diseases, Boston, United States. Directorate General for Disease Surveillance and Control, Ministry of Health, Muscat, Oman; International Society for Infectious Diseases, Boston, United States. Department of Community Medicine, Ahmadu Bello University, Zaria, Nigeria; International Society for Infectious Diseases, Boston, United States. Instituto de Microbiologia Paulo de Goes, Universidade Federal do Rio de Janeiro, Brazil; International Society for Infectious Diseases, Boston, United States. Centre d’Entomologie Médicale et Vétérinaire, CEMV – Université Alassane Ouattara, Bouaké, Côte d’Ivoire; International Society for Infectious Diseases, Boston, United States. Infectious Diseases Translational Research Program, Yong Loo Lin School of Medicine, National University of Singapore, Singapore; International Society for Infectious Diseases, Boston, United States. Department of Infectious Disease, Faculty of Medicine, Imperial College London, United Kingdom

The **International Journal of Infectious Diseases (IJID)** was inaugurated in July 1996, 25 years ago today. In the opening editorial, Dr Donald Armstrong*, then Editor-in-chief, explained that the journal’s mission was to “....communicate and disseminate new and reviewed information on infectious diseases to researchers and clinicians throughout the world..; provide information relevant to the epidemiology, clinical evaluation, diagnosis, treatment, public health and control of infections, with special emphasis on those diseases most prevalent in less-developed countries.”[1] ***IJID*** has continued to stand by the same mission that guides the Journal’s development.

Twenty-five years on, ***IJID*** has grown from a quarterly journal in the 1990s through 2003, turned bimonthly in 2004 and has been published in monthly issues, totally online, from 2010. Adoption of the Open Access model, since 2013, has changed the landscape of academic publishing in the scientific community. While some academics remain sceptical about Open Access publishing [2], it has nonetheless become the new standard for effective dissemination of scientific knowledge [3] Notably, the number of published pages in ***IJID*** has increased by more than 20-fold from 238 in 1996/1997 to 6,596 in 2020/2021. Highly cited articles were often those on emerging infections (clinical, public health and epidemiological aspects) and state-of art reviews of infectious disease management and four of the most cited papers were published in 2020 on COVID-19 (Table 1).

With the increase in emerging diseases and the re- emergence of others, advances in diagnostics, therapeutics and changes in epidemiology, an increasing diversity of infectious disease subjects have been covered by ***IJID***. Research in HIV/AIDS, childhood infections, tuberculosis and antimicrobial resistance are regularly covered in almost every issue of the Journal, while emerging infections like the new human coronaviruses such as SARS-CoV-1 and SARS-CoV-2, the avian influenzas, tropical infections (for example, malaria, Ebola, dengue, leishmaniasis, helminths) occupy journal spaces especially at times of outbreaks. The dominance of manuscripts on different aspects of the COVID-19 pandemic in a large part of 2020 was phenomenal, which reflected very rapid generation of new knowledge and the need for efficient dissemination of scientific findings. In this connection, ***IJID*** supplements and complements the role of disease control agencies on an international level.

As the official publication of the International Society for Infectious Diseases (ISID), ***IJID*** serves not just as a platform for the communication of research information but as an advocate for the fight against infectious disease threats. In response to the changing epidemiology of COVID-19, editorials have been published on the development of evidence-based strategy, including the inclusion of SARS-CoV-2 vaccine in the WHO yellow card for travel [4], and mandatory vaccination for preventing virus spread at the Tokyo Olympics [5]. ***IJID*** must continue with this role as “a new international voice for prevention and control of infectious diseases. (title of Dr Armstrong’s opening editorial) [1].

The global population will continue to face the challenges of re/emerging infections, and threats from endemic diseases both internationally and in local regions. The field of Infectious Diseases research is predicted to grow further and the demand for publication outlets will increase. To mark the 25^th^ anniversary of IJID, a ‘spin-off journal’ ***IJID Regions*** is being launched. This will showcase research with a regional focus, a perspective which has sometimes been marginalised in the ***IJID*** in the midst of fierce competition for publication space. Published work from regional research often does not just appeal to readers in selected regions but also can provide valuable lessons for those in other regions.

***IJID Regions*** aims to (a) publish high-quality, evidence-based infectious disease science of regional interest and relevance; (b) enhance the visibility of regional research with a focus on those infectious diseases that are important in under-resourced countries and settings; and (c) publish research that can be utilized by national and regional health clinicians, scientists, health systems, and public health policy makers.

***IJID*** and ***IJID Regions*** are committed to providing a home for quality research of relevance in clinical and public health practice, as well as to supporting the education of emerging scientists and practitioners.

The current editorial team is indebted to our predecessors – Editors-in-Chief Dr Donald Armstrong (1996 to 2001), Dr Jonathan Cohen (2001 to 2006), Dr William Cameron (2007-2012) and their editors for laying the foundation of ***IJID*** and preparing for the birth of ***IJID Regions***. We look forward to contributing to global infectious diseases evaluation, diagnosis, treatment and control for the next 25 years and beyond.

## Figures and Tables

**Table 1:** Ten most cited articles published in International Journal of Infectious Diseases, 2009-2020

Year	Title	First author
2020	The continuing 2019-nCoV epidemic threat of novel coronaviruses to global health — The latest 2019 novel coronavirus outbreak in Wuhan, China https://doi.org/10.1016/j.ijid.2020.01.009	Hui D.S. et al.
2020	Preliminary estimation of the basic reproduction number of novel coronavirus (2019-nCoV) in China, from 2019 to 2020: A data-driven analysis in the early phase of the outbreak https://doi.org/10.1016/j.ijid.2020.01.050	Zhao S. et al.
2020	A conceptual model for the coronavirus disease 2019 (COVID-19) outbreak in Wuhan, China with individual reaction and governmental action https://doi.org/10.1016/j.ijid.2020.02.058	Lin Q. et al.
2020	Transmission potential and severity of COVID-19 in South Korea https://doi.org/10.1016/j.ijid.2020.03.031	Shim E. et al
2016	Rapid Spread of Zika Virus in The Americas - Implications for Public Health Preparedness for Mass Gatherings at the 2016 Brazil Olympic Games https://doi.org/10.1016/j.ijid.2016.02.001	Petersen E. et al.
2010	Burden of invasive pneumococcal disease and serotype distribution among Streptococcus pneumoniae isolates in young children in Europe: impact of the 7-valent pneumococcal conjugate vaccine and considerations for future conjugate vaccines https://doi.org/10.1016/j.ijid.2009.05.010	Isaacman D.J. et al.
2010	Relative frequency of albicans and the various non-albicans Candida spp among candidemia isolates from inpatients in various parts of the world: A systematic review https://doi.org/10.1016/j.ijid.2010.04.006	Falagas M.E. et al.
2010	Fleas and flea-borne diseases https://doi.org/10.1016/j.ijid.2009.11.011	Bitam I. et al.
2009	Echinococcosis: a review https://doi.org/10.1016/j.ijid.2008.03.037	Moro P. et al.
2009	Hepatitis B and hepatitis C in Pakistan: prevalence and risk factors https://doi.org/10.1016/j.ijid.2008.06.019	Ali S.A. et al.
